# Effects of Ipriflavone-Loaded Mesoporous Nanospheres on the Differentiation of Endothelial Progenitor Cells and Their Modulation by Macrophages

**DOI:** 10.3390/nano11051102

**Published:** 2021-04-24

**Authors:** Laura Casarrubios, Alberto Polo-Montalvo, María Concepción Serrano, María José Feito, María Vallet-Regí, Daniel Arcos, María Teresa Portolés

**Affiliations:** 1 Departamento de Bioquímica y Biología Molecular, Facultad de Ciencias Químicas, Universidad Complutense de Madrid, Instituto de Investigación Sanitaria del Hospital Clínico San Carlos (IdISSC), 28040 Madrid, Spain; laura.casarrubios.molina@gmail.com (L.C.); albpolo@ucm.es (A.P.-M.); mjfeito@ucm.es (M.J.F.); 2 Instituto de Ciencia de Materiales de Madrid (ICMM), Consejo Superior de Investigaciones Científicas (CSIC), 28049 Madrid, Spain; mc.terradas@csic.es; 3 Departamento de Química en Ciencias Farmacéuticas, Facultad de Farmacia, Universidad Complutense de Madrid, Instituto de Investigación Sanitaria Hospital 12 de Octubre i + 12, Plaza Ramón y Cajal s/n, 28040 Madrid, Spain; 4 CIBER de Bioingeniería, Biomateriales y Nanomedicina, CIBER-BBN, 28040 Madrid, Spain

**Keywords:** endothelial progenitor cells, macrophages, mesoporous nanospheres, vascular endothelial growth factor receptor 2, ipriflavone, endocytosis

## Abstract

Angiogenic biomaterials are designed to promote vascularization and tissue regeneration. Nanoparticles of bioactive materials loaded with drugs represent an interesting strategy to stimulate osteogenesis and angiogenesis and to inhibit bone resorption. In this work, porcine endothelial progenitor cells (EPCs), essential for blood vessel formation, were isolated and characterized to evaluate the in vitro effects of unloaded (NanoMBGs) and ipriflavone-loaded nanospheres (NanoMBG-IPs), which were designed to prevent osteoporosis. The expression of vascular endothelial growth factor receptor 2 (VEGFR2) was studied in EPCs under different culture conditions: (a) treatment with NanoMBGs or NanoMBG-IPs, (b) culture with media from basal, M1, and M2 macrophages previously treated with NanoMBGs or NanoMBG-IPs, (c) coculture with macrophages in the presence of NanoMBGs or NanoMBG-IPs, and (d) coculture with M2d angiogenic macrophages. The endocytic mechanisms for nanosphere incorporation by EPCs were identified using six different endocytosis inhibitors. The results evidence the great potential of these nanomaterials to enhance VEGFR2 expression and angiogenesis, after intracellular incorporation by EPCs through clathrin-dependent endocytosis, phagocytosis, and caveolae-mediated uptake. The treatment of EPCs with basal, M1, and M2 macrophage culture media and EPC/macrophage coculture studies also confirmed the angiogenic effect of these nanospheres on EPCs, even in the presence of phagocytic cells.

## 1. Introduction

The vascular system plays an essential role in the efficient supply of oxygen and nutrients needed to ensure the development of all tissues. Vascular structures can be formed through vasculogenesis (*de novo* blood vessels created during embryonic development) or angiogenesis (new vessels from a pre-existing vascular network) [1,2]. It is important to highlight that different signals between vascular and nonvascular cells are essential to initiate the angiogenic events that are required for regenerative processes [3,4]. In particular, macrophages that infiltrate the damaged tissue secrete growth factors and other molecules to stimulate angiogenesis depending on their phenotype and activation state [5,6]. It is well known that macrophages are influenced by multiple microenvironmental stimuli able to induce macrophage polarization toward M1 proinflammatory or M2 reparative phenotypes [7]. M1 macrophages are mainly induced by IFN-γ and bacterial lipopolysaccharide (LPS), producing higher levels of proinflammatory cytokines and reactive oxygen species (ROS) [8]. In contrast, M2 macrophages are induced by cytokines such as IL-4, IL-10, IL-13, IL-21, and IL-33 and have an anti-inflammatory cytokine profile. Functionally, M2 macrophages possess proangiogenic properties and promote tissue repair and wound healing [9]. Depending on the activating stimulus, M2 macrophages can be divided into four subsets: M2a, M2b, M2c, and M2d [10], which are induced by different cytokines and produce specific molecules with different activities [8]. The fourth type of M2 macrophages, M2d, is induced by agonists that target Toll-like receptors (TLRs) through the adenosine receptor, followed by the suppression of proinflammatory cytokine production and the induction of secretion of both anti-inflammatory cytokines and vascular endothelial growth factor (VEGF) with proangiogenic properties [11,12].

VEGF acts on endothelial cells inducing angiogenesis [13] and regulates vascular permeability [14]. The secretion of this factor by macrophages plays an important role in wound healing, as well as in chronic inflammation and cancer [12]. Among the known vascular endothelial growth factors (VEGFs) and receptors (VEGFRs), VEGF-A regulates angiogenesis and vascular permeability by activating VEGFR1 and VEGFR2 receptors, with VEGFR2 being the major signal transducer for angiogenesis through the PLCγ–PKC–MAPK pathway [15]. VEGF-A also stimulates bone repair by promoting angiogenesis, maturation of osteoblasts, ossification, and bone turnover [16]. Thus, osteogenesis is a process coupled to vascularization during bone development and growth [17]. For this reason, angiogenic biomaterials are designed to promote vascularization and optimize bone regeneration [18,19,20,21]. In this context, nanoparticles of bioactive materials loaded with different drugs represent an interesting strategy to promote bone regeneration by stimulating osteogenesis and angiogenesis and inhibiting bone resorption [22,23]. Among the drugs explored with this aim, ipriflavone (IP) prevents osteoporosis by inhibiting the activity of osteoclasts [23,24] and stimulating the differentiation of pre-osteoblasts into mature osteoblasts [25]. The administration of the drug with nanoparticles allows significantly reducing the quantity of drug required to carry out the desire effect.

Since endothelial progenitor cells (EPCs) are involved in blood vessel formation, essential for tissue regeneration, porcine EPCs have been used in this work to evaluate their response to ipriflavone-loaded mesoporous bioactive nanospheres, designed to stimulate osteogenesis. These nanospheres (NanoMBGs) have shown excellent in vitro behavior in previous studies with bone cells [23,25]. The expression of VEGFR2, directly related to angiogenesis, has been analyzed in EPCs treated with these nanospheres considering the possible modulating role of macrophages, depending on their phenotype and activation state. With this objective, the actions of NanoMBGs without or with ipriflavone (NanoMBG-IPs) and macrophage mediators on angiogenesis were evaluated by assessing VEGFR2 expression, under different conditions: (a) EPCs in monoculture treated with NanoMBGs or NanoMBG-IPs, (b) EPCs treated with conditioned media from basal, M1, and M2 macrophages previously treated with NanoMBGs or NanoMBG-IPs, (c) EPCs cocultured with macrophages in the presence of NanoMBGs or NanoMBG-IPs, and (d) EPCs cocultured with M2d angiogenic macrophages. On the other hand, the endocytic mechanisms via which these nanospheres are incorporated by EPCs were identified using six endocytosis inhibitors (i.e., wortmannin, genistein, cytochalasin B, cytochalasin D, phenylarsine oxide, and chlorpromazine) before the addition of NanoMBGs labeled with fluorescein isothiocyanate (FITC-NanoMBGs). Overall, the present work will contribute to the knowledge of the incorporation mechanisms of these ipriflavone-loaded nanospheres into EPCs to promote tissue regeneration and the modulatory role played by macrophages as main cells of the innate immune response to this kind of nanomaterial.

## 2. Materials and Methods

### 2.1. Preparation, Characterization, Labeling with FITC, and Loading with Ipriflavone of Mesoporous Nanospheres

Mesoporous nanospheres (NanoMBGs) with nominal chemical composition 75 SiO_2_/20 CaO/5 P_2_O_5_ (mol.%) were synthesized, characterized, and labeled with fluorescein isothiocyanate (FITC-NanoMBGs) as previously described [23,25]. Briefly, 80 mg of poly(styrene)-*block*-poly(acrylic acid) and 160 mg of hexadecyl trimethyl ammonium bromide (CTAB) were dissolved in tetrahydrofurane (16 mL) and water/ammonia (74 mL/2.4 mL), respectively, and mixed together under vigorous magnetic stirring. Subsequently, 25 μL of triethylphosphate (TEP) and 0.52 mL of tetraethoxysilane (TEOS) previously dissolved in ethanol were added dropwise under stirring. Then, 125 mg of Ca(NO_3_)_2_·4H_2_O in 1.6 mL of water was also added and stirred for 10 min more. After stirring for 24 h, the resulting product was collected by centrifugation and treated at 550 °C to remove the organic template.

The nanoparticles were subsequently labeled with FITC by dissolving 0.6 mg of FITC and 44.3 μL of aminopropyl triethoxysilane (APTS) in 0.5 mL of ethanol. This solution was added dropwise to 50 mg of nanoparticles previously suspended in toluene and left to react at 80 °C for 12 h under nitrogen atmosphere.

Ipriflavone-loaded nanoparticles (NanoMBG-IPs) were prepared by dissolving 250 mg of ipriflavone in 3 mL of acetone. Thereafter, 100 mg of previously dried nanoparticles treated at 100 °C for 24 h were added to the solution and gently stirred in an incubator at 37 °C with a rotation speed of 100 rpm for 12 h. After this period, the nanoparticles were collected by filtration under vacuum using a polyamide filter.

For morphological characterization of nanoparticles, scanning electron microscopy (SEM) and transmission electron microscopy (TEM) images were collected with a JEOL F-6335 microscope and a JEOL-1400 microscope (JEOL, Tokyo, Japan), respectively. Thermogravimetric analysis was carried out with a TG/DTA Seiko SSC/5200 thermobalance. The thermogram was obtained from 100 °C to 650 °C using a platinum crucible and α-Al_2_O_3_ as a reference.

### 2.2. Obtention and Culture of Endothelial Progenitor Cells (EPCs)

Endothelial progenitor cells (EPCs) were obtained as previously described [26,27]. Briefly, whole pig blood was diluted (1:1) in phosphate-buffered saline (PBS) with 0.1% of bovine serum albumin (BSA) and 0.6% of sodium citrate. Mononuclear cells (MNCs) were isolated using a density gradient formed with Histopaque-1077 solution (Sigma–Aldrich Corporation, St. Louis, MO, USA) in AccuspinTM tubes (Sigma–Aldrich Corporation, St. Louis, MO, USA). The samples were centrifuged at 800× *g* for 30 min at room temperature. The MNC layer was carefully collected and seeded in Endothelial Growth Medium (EGM-2, Sigma–Aldrich Corporation, St. Louis, MO, USA) in F75 polystyrene culture flasks (Corning Inc., Corning, NY, USA) at a density of 2–3 × 10^5^ cells/cm^2^ under a CO_2_ (5%) atmosphere and at 37 °C. The culture medium was replaced at 96 h and then every 48 h until confluence. Confluent cultures of EPCs were maintained in EGM-2 until cells acquired the cobblestone morphology characteristic of mature endothelial cells. After confirmation of their endothelial phenotype as described below (Section 2.3), cells were cultured in EGM-2 with or without nanospheres for several times in different culture plates and at different cell densities according to the type of experiment: (a) in 12-well culture plates (Corning Inc., Corning, NY, USA) at a density of 10^5^ cells/well during 24 h for the subsequent incorporation of FITC-labeled NanoMBGs for 30, 60, and 90 min (Section 2.4) and (b) in six-well culture plates (Corning Inc., Corning, NY, USA) at a density of 15 × 10^3^ cells/well during 10 days for evaluation of VEGFR2 expression in different conditions (Section 2.5, Section 2.6 and Section 2.7).

### 2.3. Phenotypic Characterization of EPCs

After the obtention of EPCs as described above, the following markers were evaluated to assess their differentiation toward a mature endothelial phenotype after 23 and 30 days of culture in EGM-2:

- CD31, transmembrane receptor also known as platelet endothelial cell adhesion molecule-1, PECAM-1 [28];
- CD34, transmembrane cell surface glycoprotein selectively expressed within the hematopoietic system on stem and progenitor cells [29];
- eNOS, endothelial nitric oxide synthase, responsible for the endothelial production of NO [30];
- vWF, von Willebrand factor, glycoprotein produced by endothelial cells and megakaryocytes [31];
- VEGFR2, VEGF receptor 2, whose expression is restricted to endothelial cells, monocytes, and hematopoietic precursors [32].

For flow cytometry studies, 10^6^ cells were used to carry out the identification of each of these markers by immunostaining with corresponding antibodies. The antibodies used in this study were as follows: anti-CD31 (TLD-3A12, ab64543, Abcam, Cambridge, UK), anti-CD34 (EP373Y, ab81289, Abcam, Cambridge, UK), anti-eNOS (M221, ab76198, Abcam, Cambridge, UK), anti-vWF (ab6994, Abcam, Cambridge, UK), and anti-VEGFR2 (ab2349, Abcam, Cambridge, UK). Two secondary antibodies were used, one conjugated with DyLight 633 (IgG (H + L) goat anti-rabbit DyLight^®^ 633, Invitrogen, Carlsbad, CA, USA) and the other with Alexa 488 (IgG (H + L) Highly cross-adsorbed goat anti-mouse Alexa Fluor^®^ Plus 488, Invitrogen, Carlsbad, CA, USA). All antibodies were prepared in normal goat serum (NGS) at 2% for use. The final antibody concentrations were 10 µg/mL for flow cytometry and 5 µg/mL for confocal microscopy, unless specifically prescribed by the supplier. In the case of intracellular markers (eNOS and vWF), the cells were previously permeabilized with 0.25% saponin for 10 min at 4 °C before adding the antibodies, and saponin at 0.25% was maintained at all subsequent steps of immunolabeling. To prevent nonspecific binding, cells were incubated in 10% PBS/NGS for 10 min at room temperature. After centrifugation, 300 µL of the corresponding primary antibody was added to the pellet, incubating the tubes for 30 min at room temperature in darkness. Then, 700 µL of 1% PBS/BSA was added for 10 min at room temperature for washing and, after centrifugation, the pellet was incubated with 300 µL of the corresponding secondary antibody for 30 min at room temperature in darkness. Finally, the pellet was washed with 700 µL of 1% PBS/BSA for 10 min at room temperature, centrifuged, and resuspended in 300 µL of PBS. The resulting EPC suspensions labeled with the different antibodies were analyzed in a FACScalibur Becton Dickinson flow cytometer. The fluorescence of Alexa Fluor 488 was excited at 488 nm and measured at 519 nm. The fluorescence of DyLight 633 was excited at 638 nm and measured at 658 nm.

For confocal microscopy studies, EPCs were cultured on glass coverslips under the above conditions, fixed with 0.5 mL of 4% paraformaldehyde for 10 min at room temperature and washed with PBS three times. For intracellular labeling, cell membranes were permeabilized with 0.5 mL of 70% ethanol at 4 °C for 10 min and washed with PBS three times. To avoid nonspecific binding, samples were incubated with 0.5 mL of 10% PBS/NGS for 10 min at room temperature and washed with PBS three times. Then, 50 µL of the corresponding primary antibody was added in 2% PBS/NGS at a final concentration of 5 µg/mL for 1 h at room temperature. After that, samples were washed three times with 0.5 mL of 2% PBS/NGS, and 50 µL of the corresponding secondary antibody was added to a final concentration of 5 µg/mL. After washing three times with 0.5 mL of PBS, 3 µM 4′-6-diamidine-2′-phenylindole (DAPI, Molecular Probes, Eugene, OR, USA) was added for 5 min, and the glass coverslips were mounted on slides with ProLongTM Gold (Invitrogen, Carlsbad, CA, USA). Samples were then observed on a Leica SP2 AOBS multiphoton confocal laser microscope. DAPI fluorescence was excited at 405 nm and measured at 420–480 nm. The fluorescence of Alexa FluorTM was excited at 488 nm and measured at 519 nm. The fluorescence of DyLightTM 633 was excited at 638 nm and measured at 658 nm.

### 2.4. Analysis of the Incorporation of FITC-Labeled NanoMBGs by EPCs and Identification of Endocytic Mechanisms

EPCs cultured in EGM-2 for 24 h were exposed to 50 µg/mL of FITC-NanoMBGs for 30, 60, and 90 min. After that, cells were harvested with trypsin-EDTA (0.25%), and FITC-NanoMBG incorporation was quantified through flow cytometry. The FITC-NanoMBG fluorescence was detected in a FACScalibur Becton Dickinson flow cytometer with a 530/30 filter, exciting the sample at 488 nm. Data acquisition and flow cytometric analysis conditions were set through negative and positive controls using the CellQuest Program of Becton Dickinson and maintained for all measurements. For each sample, 10^4^ cells were analyzed to ensure a correct statistical significance.

For confocal microscopy studies, EPCs were cultured on circular glass coverslips in the presence of 50 µg/mL of FITC-NanoMBGs for 24 h. Afterward, cells were fixed with paraformaldehyde (3.7%) and permeated with 500 μL of Triton-X100 (0.1% in PBS). After 20 min of incubation with BSA (1% in PBS), samples were stained with rhodamine–phalloidin 1:40 (100 μL), washed with PBS, and stained with 100 μL of 3 μM DAPI. Finally, samples were observed by using an Olympus FV1200 confocal laser scanning microscope. FITC fluorescence was excited at 488 nm and measured at 491–586 nm. Rhodamine fluorescence was excited at 546 nm and detected at 600–620 nm. DAPI fluorescence was excited at 405 nm and detected at 420–480 nm.

To identify the endocytic mechanisms via which FITC-NanoMBGs were incorporated by EPCs, the treatment with six specific endocytosis inhibitors was carried out before adding the nanoparticles, maintaining the cells for 2 h under the conditions described above. The endocytosis inhibitors were as follows: 23 μM wortmannin (Enzo Life Sciences, Barcelona, Spain), 3.7 μM genistein (Enzo Life Sciences, Barcelona, Spain), 20 μM cytochalasin B (MP Biomedicals, Eschwege, Germany), 4 μM cytochalasin D (MP Biomedicals, Eschwege, Germany), 3.7 μM phenylarsine oxide (PAO, Sigma–Aldrich Corporation, St. Louis, MO, USA), and 30 μM chlorpromazine (Enzo Life Sciences, Barcelona, Spain). Then, 50 µg/mL of FITC-NanoMBGs were added to the medium and cells were maintained for 30 min under the conditions described above. Finally, cells were collected with trypsin-EDTA (0.25%), and the FITC-NanoMBG incorporation in each case was quantified by flow cytometry. Controls without inhibitors were carried out in parallel. The dose of each inhibitor was chosen according to previous studies [25,33,34,35,36,37].

### 2.5. Evaluation of VEGFR2 Expression in EPCs after Treatment with NanoMBGs and NanoMBG-IPs

EPCs were cultured in the presence of 50 μg/mL of unloaded and IP-loaded nanospheres (NanoMBGs and NanoMBG-IPs, respectively) for 10 days under the conditions described above, refreshing the culture medium with nanospheres every 3 days. To evaluate the intracellular action of ipriflavone on the differentiation of EPCs, cells were harvested with trypsin-EDTA (0.25%), and VEGFR2 expression was quantified by flow cytometry as a specific marker of EPC differentiation after immunostaining with the corresponding antibody as described above (Section 2.3).

### 2.6. Culture, M1/M2 Stimulation, and Treatment of RAW 264.7 Macrophages with NanoMBGs and NanoMBG-IPs. Treatment of EPCs with Macrophage Conditioned Media to Evaluate VEGFR2 Expression

RAW 264.7 macrophages (American Type Culture Collection, ATCC) were seeded in six-well culture plates (Corning Inc., Corning, NY, USA), at a density of 10^5^ cells/mL, in 2 mL of Dulbecco’s modified Eagle medium (DMEM) supplemented with 10% fetal bovine serum (FBS, Gibco, BRL), 1 mM l-glutamine (BioWhittaker Europe, Verviers, Belgium), penicillin (200 μg/mL, BioWhittaker Europe, Belgium), and streptomycin (200 μg/mL, BioWhittaker Europe, Belgium) at 37 °C under a CO_2_ (5%) atmosphere. Cells were treated for 24 h in the absence or in the presence of 50 μg/mL of unloaded and IP-loaded nanospheres (NanoMBGs and NanoMBG-IPs, respectively) in basal or stimulated conditions in order to induce the polarization of macrophages toward M1 or M2 phenotypes. To induce the M1 phenotype, macrophages were culture in the presence of *E. coli* lipopolysaccharide (LPS, 250 ng/mL, Sigma-Aldrich Corporation, St. Louis, MO, USA) [38]. To induce the M2 phenotype, macrophages were culture in the presence of interleukin-10 (IL-10, 40 ng/mL, BioLegend, San Diego, CA, USA) [39]. After these treatments, media were aspirated and kept at −20 °C until use. EPCs were cultured in six-well culture plates (15 × 10^3^ cells/well) and treated for 10 days with these conditioned media from basal, M1, and M2 macrophages to evaluate their effects on VEGFR2 expression by flow cytometry as described above (Section 2.3). The conditioned medium was changed every 3 days.

### 2.7. Coculture of EPCs and RAW 264.7 Macrophages in the Presence of NanoMBGs and NanoMBG-IPs. Evaluation of VEGFR2 Expression in EPCs and CD206 Expression in RAW 264.7 Macrophages

**Scheme 1:**
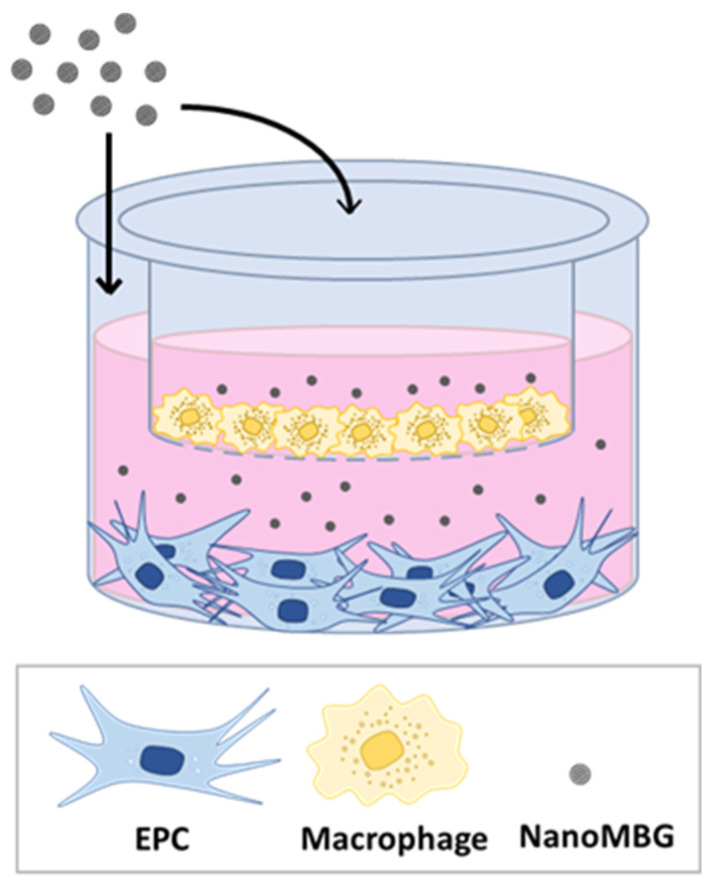
Cocultures of endothelial progenitor cells (EPCs) and RAW 264.7 macrophages in the presence of 50 μg/mL of NanoMBG nanospheres with or without ipriflavone.

EPCs were seeded in six-well culture plates at a density of 15 × 10^3^ cells/well. Simultaneously, RAW 264.7 cells were seeded in transwell inserts (0.4 μm pore size, Corning Inc., Corning, NY, USA) at a density of 15 × 10^3^ cells/transwell in 1.3 mL of the same culture medium and placed into the six-well culture plates containing seeded EPCs (Scheme 1). For coculture, we used a medium composed of 50% DMEM and 50% EGM-2, having previously verified that both cell types proliferate adequately. These cocultures were carried out in the presence or the absence of 50 μg/mL of NanoMBGs without or with ipriflavone (NanoMBG-IPs) for 7 days at 37 °C under a CO_2_ (5%) atmosphere. In parallel, EPCs and macrophages were cultured alone in wells and transwell inserts, respectively, as controls. After coculturing, EPCs and macrophages were washed with PBS, harvested using 0.25% trypsin-EDTA solution and cell scrapers, respectively, centrifuged at 310× *g* for 10 min, and resuspended in PBS for the analysis of the expression of VEGFR2 in EPCs and CD206 in RAW 264.7 macrophages by flow cytometry. VEGFR2 expression, as a specific marker of EPCs differentiation into mature endothelium, was evaluated by flow cytometry as described above (Section 2.3). CD206 expression, as a specific marker of M2 macrophage phenotype [40], was quantified by flow cytometry as described below. After detachment and centrifugation, macrophages were incubated in 45 µL of staining buffer (PBS, 2.5% FBS Gibco, BRL and 0.1% sodium azide, Sigma-Aldrich Corporation, St. Louis, MO, USA) with 5 µL of normal mouse serum inactivated for 15 min at 4 °C in order to block the Fc receptors on the macrophage plasma membrane, before adding the primary antibody, and to prevent nonspecific binding. Then, cells were incubated with FITC anti-mouse CD206 (15 µg/mL, BioLegend, San Diego, CA, USA) for 30 min in the dark. Labeled cells were analyzed using a FACSCalibur flow cytometer. FITC fluorescence was excited at 488 nm and measured with a 530/30 band pass filter.

The effects of NanoMBGs and NanoMBG-IPs on the expression of VEGFR2 and CD206 in EPC/macrophage cocultures were compared with the expression of these specific markers when EPCs were cocultured with M2d angiogenic macrophages. M2d angiogenic macrophages were obtained from RAW 264.7 macrophages treated with 5’-*N*-ethyl-carboxamido-adenosine (NECA, 1 μM, MedChemExpress, Monmouth Junction, NJ, USA)/*E. coli* LPS (100 ng/mL, Sigma-Aldrich Corporation, St. Louis, MO, USA) [12].

### 2.8. Statistics

Results were expressed as the means of three identical experiments with their corresponding standard deviations, analyzed using the 22^nd^ version of Statistical Package for the Social Sciences (SPSS). Statistical comparisons were carried out by one-way analysis of variance (ANOVA), and Scheffé and Games–Howell tests were used for post-hoc analysis of differences between study groups, considering *p* < 0.05 as statistically significant.

## 3. Results and Discussion

### 3.1. Characterization of Mesoporous Nanospheres

**Figure 1:**
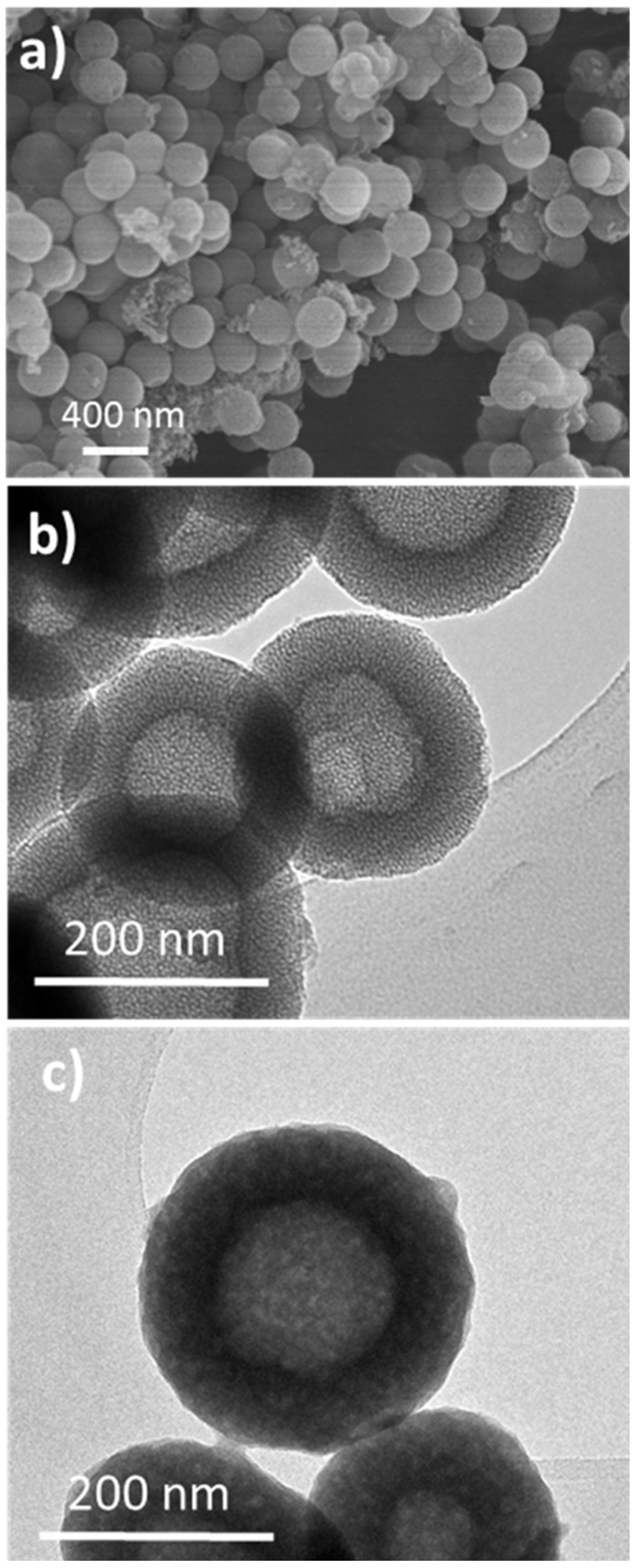
Representative SEM micrograph obtained from Nano-MBGs (**a**). Representative TEM images of Nano-MBGs before loading with ipriflavone (**b**) and after loading with iproflavone (Nano-MBG-IPs) (**c**).

Figure 1a,b show representative SEM and TEM images obtained from Nano-MBG samples, i.e., before loading with ipriflavone. The particles showed an external spherical morphology (Figure 1a) and a mesoporous structure consisting of a hollow core surrounded by a radial mesoporous shell (Figure 1b). Subsequent TEM observations after the loading process indicated a significant loss of mesoporous structure because of drug loading process (Figure 1c). The amount of loaded ipriflavone was 19.3% ± 1.7% (wt.%) as determined by thermogravimetric analysis, pointing out that the mesoporous structure introduced by the organic templates was appropriate for the use of Nano-MBG particles as matrices for loading this antiosteoporotic drug.

### 3.2. Differentiation and Phenotypic Characterization of EPCs

EPCs are considered a promising cell strategy for tissue engineering applications due to their highly proliferative and antithrombogenic behavior [41,42]. This cell type can be easily obtained from peripheral blood [26,27], and it is very appropriate as an in vitro experimental model for the study of the angiogenic potential of different biomaterials [19,21,43,44]. In this work, after isolating EPCs from porcine peripheral blood and culturing for 23 and 30 days in EGM-2 differentiation medium, the expression of CD31, CD34, VEGFR2, eNOS, and vWF, as endothelial phenotype markers, was analyzed by flow cytometry.

**Figure 2:**
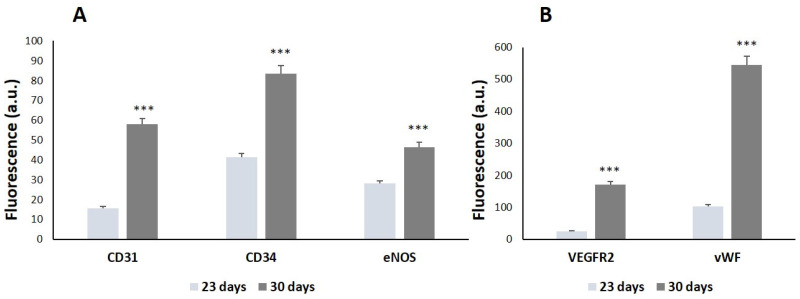
Phenotypic characterization of EPCs in culture. The expression of (**A**) CD31, CD34, and eNOS and (**B**) VEGFR2 and vWF, was evaluated in EPCs as endothelial phenotype markers by immunofluorescence labeling and flow cytometry after 23 and 30 days of differentiation. In each sample, 10,000 cells were analyzed. Statistical significance: *** *p* < 0.005.

As observed in Figure 2, the expression of the endothelial phenotype markers significantly increased in a time-dependent manner, thus indicating the correct differentiation of the EPCs toward a mature endothelial phenotype. When comparing the values obtained at 23 and 30 days, VEGFR2 and vWF (Figure 2B) showed more significant increases in comparison to the other markers CD31, CD34, and eNOS (Figure 2A). According to these results and considering that the expression of VEGFR2 is directly related to angiogenesis, VEGFR2 was chosen as a reference marker to assess the potential angiogenic effect of ipriflavone-loaded nanospheres (NanoMBG-IPs) on EPCs after 30 days of differentiation, as well as the modulating role of M1 and M2 macrophages on them.

### 3.3. Incorporation of FITC-Labeled NanoMBGs by EPCs and Endocytic Mechanisms

**Figure 3:**
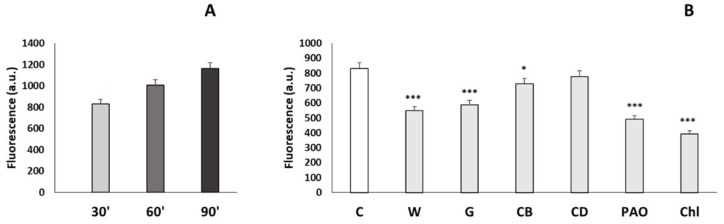
(**A**) Incorporation of FITC-NanoMBGs by EPCs evaluated by flow cytometry. Fluorescence intensity of EPCs with intracellular FITC-NanoMBGs after treatment with 50 µg/mL for different times (30, 60, and 90 min). Statistical significance: *** *p* < 0.005. (**B**) Effects of specific endocytosis inhibitors on FITC-NanoMBG incorporation by EPCs. EPCs were treated for 2 h with each inhibitor before treatment with 50 µg/mL FITC-NanoMBGs for 30 min. FITC-NanoMBG incorporation in each case was quantified by flow cytometry. Control cells without inhibitors were carried out in parallel. C = control cells, W = cells treated with wortmannin, G = cells treated with genistein, CB = cells treated with cytochalasin B, CD = cells treated with cytochalasin D, PAO = cells treated with phenylarsine oxide, Chl = cells treated with chlorpromazine. Statistical significance: * *p* < 0.05, *** *p* < 0.005.

**Figure 4:**
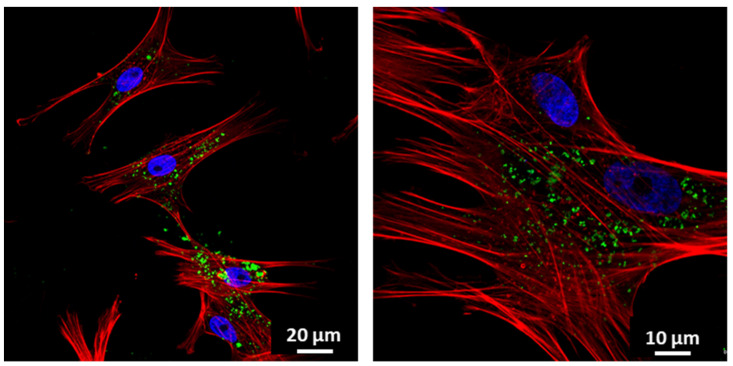
Incorporation of FITC-NanoMBGs by EPCs observed by confocal microscopy. Images of EPCs after 24 h of incubation with 50 μg/mL of FITC-NanoMBGs. Nuclei were stained with DAPI (blue), F-actin filaments were stained with rhodamine-phalloidin (red), and FITC-NanoMBGs are observed in green.

To evaluate the incorporation of NanoMBGs by EPCs, these nanospheres were labeled with FITC. EPCs were cultured for 24 h and then treated with 50 µg/mL of FITC-NanoMBGs for 30, 60, and 90 min in order to quantify the amount of intracellular uptake of this nanomaterial by flow cytometry. Figure 3A shows the intracellular fluorescence intensity of EPCs after this treatment, evidencing a fast and time-dependent uptake of FITC-NanoMBGs, in agreement with previous studies carried out with these nanospheres in other cell types [25,45]. Moreover, confocal microscopy studies were carried out after treatment of EPCs with 50 µg/mL of FITC-NanoMBGs for 24 h to observe the intracellular uptake of this nanomaterial and to evaluate if its incorporation could damage the cytoskeleton structure. Figure 4 shows the abundance of nanospheres into the cytoplasm of EPCs and the integrity of their morphology without cytoskeleton alterations after the uptake.

On the other hand, to identify the endocytic mechanisms via which these nanospheres are incorporated by EPCs, six endocytosis inhibitors (i.e., wortmannin, genistein, cytochalasin B, cytochalasin D, phenylarsine oxide, and chlorpromazine) were used before the addition of FITC-NanoMBGs. Since these agents affect different proteins, thus blocking specific endocytic mechanisms, their use indirectly provides information on the pathways that could be involved in the entry of these nanospheres. In this sense, when the inhibitor used decreases the uptake of nanospheres, the blocked mechanism constitutes an entry pathway. In contrast, if the inhibitor does not decrease the incorporation of the nanospheres, that specific mechanism that has been blocked by the inhibitor is not involved in the nanosphere entry. The dose of each inhibitor was chosen according to previous studies [25,33,34,35,36,37]. As it can be observed in Figure 3B, all inhibitors except cytochalasin D significantly decreased the incorporation of FITC-NanoMBGs, demonstrating that this nanomaterial is incorporated into EPCs via several uptake mechanisms.

Specifically, wortmannin inhibits phosphoinositide 3-kinase (PI3K) and phosphoinositide 4-kinase (PI4K) [33], which play important roles in cell adhesion, proliferation, motility, apoptosis, and cytoskeletal organization, impacting the phagocytosis mechanisms [46]. On the other hand, genistein produces inhibition of Src tyrosine kinases and affects clathrin-independent endocytosis mediated by caveolae [36]. Since wortmannin and genistein significantly decreased the incorporation of FITC-NanoMBGs by EPCs (*p* < 0.005), we can conclude that phagocytosis and caveolae-mediated uptake are mechanisms involved in the uptake of these nanospheres by this cell type.

Cytochalasins B and D inhibit macropinocytosis via actin polymerization blockage, preventing microfilament action [35,36]. These inhibitors slightly decreased the nanomaterial uptake, but only the effect of cytochalasin B was significant at these experimental conditions (*p* < 0.05). Similar results were obtained in a previous study, when the effect of these inhibitors on FITC-NanoMBG incorporation by pre-osteoblasts was analyzed [25]. Phenylarsine oxide (PAO), an inhibitor of clathrin-dependent endocytosis [34,47,48], and chlorpromazine, also an inhibitor of clathrin-mediated endocytosis [35], induced more significantly pronounced effects than the other agents, evidencing that clathrin-dependent endocytosis is the main entry mechanism of FITC-NanoMBGs into EPCs.

### 3.4. Effects of NanoMBGs and NanoMBG-IPs on VEGFR2 Expression in EPCs. Modulating Role of M1 and M2 Macrophages

**Figure 5:**
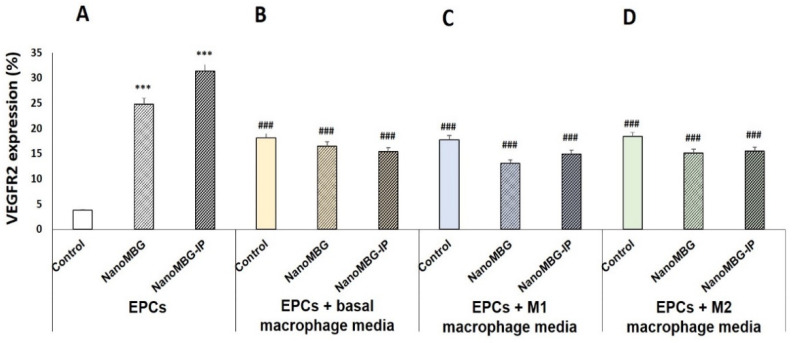
Effects of unloaded (NanoMBGs) and ipriflavone-loaded nanospheres (NanoMBG-IPs) on VEGFR2 expression in EPCs and modulating role of M1 and M2 macrophages. Percentages of VEGFR2^+^ EPCs were analyzed by flow cytometry after 10 days of treatment under the following conditions: (**A**) direct treatment with 50 µg/mL of either NanoMBGs or NanoMBG-IPs; (**B**) treatment with culture media of basal macrophages previously cultured for 24 h in the absence or in the presence of 50 μg/mL of NanoMBGs or NanoMBG-IPs; (**C**) treatment with culture media of *E. coli* LPS-stimulated M1 macrophages previously cultured for 24 h in the absence or in the presence of 50 μg/mL of NanoMBGs or NanoMBG-IPs; (**D**) treatment with culture media of IL-10-stimulated M2 macrophages previously cultured for 24 h in the absence or in the presence of 50 μg/mL of NanoMBGs or NanoMBG-IPs. Statistical significance: *** *p* < 0.005 (comparisons with control EPCs directly treated with either NanoMBGs or NanoMBG-IPs), ### *p* < 0.005 (comparison with EPCs in the same culture conditions without macrophage conditioned medium).

After demonstration of an efficient intracellular incorporation of FITC-NanoMBGs into EPCs by flow cytometry and confocal microscopy and elucidation of the entry pathways of this nanomaterial into these cells, the effect of unloaded (NanoMBGs) and ipriflavone-loaded nanospheres (NanoMBG-IPs) on the differentiation process of EPCs was analyzed by studying VEGFR2 expression as a specific marker of endothelial differentiation. As it can be observed in Figure 5A, the uptake of NanoMBGs and NanoMBG-IPs induced significant increases in the percentage of VEGFR2^+^ EPCs compared to control cells after 10 days of treatment with 50 μg/mL of these nanomaterials. These results evidence the ability of these mesoporous nanospheres, even without the drug, to stimulate VEGFR2 expression in EPCs and highlight their potential action on angiogenesis. This effect was more pronounced with NanoMBG-IPs than with NanoMBGs, thus indicating the efficient intracellular release of IP from these nanospheres and its positive in vitro effect on the differentiation of EPCs toward more mature endothelial phenotypes. These data are in consonance with results obtained by us and other authors in relation to the stimulation of osteogenesis and angiogenesis by delivering Si ions and functional drugs from mesoporous silica nanospheres [23,25,49]. Moreover, since macrophages secrete growth factors and other molecules to stimulate angiogenesis depending on their phenotype and activation state [5,6], we wanted to assess the modulating role of conditioned media from basal, M1, and M2 macrophages on the differentiation of EPCs and the effects of the previous 24 h treatment of these macrophages with 50 μg/mL of NanoMBGs and NanoMBG-IPs. Figure 5B–D show the percentages of VEGFR2^+^ EPCs after 10 days in the presence of the following macrophage conditioned media: (B) treatment with culture media from basal macrophages previously cultured for 24 h in the absence or in the presence of 50 μg/mL of NanoMBGs or NanoMBG-IPs; (C) treatment with culture media from *E. coli* LPS-stimulated M1 macrophages previously cultured for 24 h in the absence or in the presence of 50 μg/mL of NanoMBGs or NanoMBG-IPs; (D) treatment with culture media from IL-10-stimulated M2 macrophages previously cultured for 24 h in the absence or in the presence of 50 μg/mL of NanoMBGs or NanoMBG-IPs.

The treatment of EPCs for 10 days with culture media from basal, M1, and M2 macrophages previously cultured for 24 h in the absence of nanospheres induced significant increases in the percentage of VEGFR2^+^ EPCs (control bar in Figure 5B–D) compared to control cells (control bar in Figure 5A). However, these increases were less pronounced than the increments produced by the direct treatment of EPCs with NanoMBGs or NanoMBG-IPs (NanoMBGs bar and NanoMBG-IPs bar in Figure 5A). The similar effect of culture media from control macrophages in basal conditions or in both stimulated conditions (stimulation towards M1 or towards M2) demonstrates that the modulatory effect of macrophages on EPCs differentiation is related to macrophage intervention *per se*, independently of the existence or not of stimuli and the type of stimulus used in these assays. When we investigated the effect of conditioned media from macrophages previously treated with both NanoMBGs and NanoMBG-IPs (NanoMBGs bar and NanoMBG-IPs bar in Figure 5B–D), similar results were obtained, thus indicating that the pretreatment of macrophages with these nanospheres under basal or stimulated conditions does not change the effect induced by macrophage-released mediators on VEGFR2 expression. Previous studies evidenced that these NanoMBGs did not induce macrophage polarization toward the M1 proinflammatory phenotype, favoring the M2 reparative phenotype and increasing the macrophage response capability against stimuli such as LPS and IL-4 [23].

### 3.5. Effects of NanoMBGs and NanoMBG-IPs on VEGFR2 and CD206 Expression in Cocultured EPCs and RAW 264.7 Macrophages, Respectively

Next, we analyzed the effect of NanoMBGs and NanoMBG-IPs in cocultures of EPCs and RAW 264.7 macrophages, so that they could maintain communication with each other. These cocultures were carried out in the presence or the absence of 50 μg/mL of NanoMBGs or NanoMBG-IPs for 7 days. After coculture, EPCs and macrophages were harvested separately for the analysis of VEGFR2 and CD206 expression in EPCs and RAW 264.7 macrophages, respectively. EPCs and macrophages cultured alone served as controls. The expression of these specific markers when EPCs were cocultured with M2d angiogenic macrophages obtained from RAW 264.7 macrophages treated with 1 μM NECA, and 100 ng/mL of *E. coli* LPS was also used for comparison.

**Figure 6:**
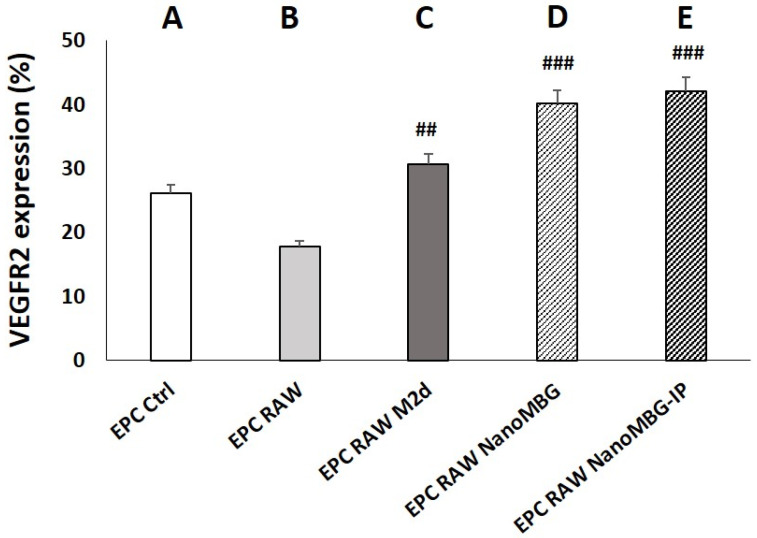
Effects of unloaded (NanoMBGs) and ipriflavone-loaded nanospheres (NanoMBG-IPs) on VEGFR2 expression in EPCs cocultured with RAW 264.7 macrophages. Percentages of VEGFR2^+^ EPCs after 7 days under the following conditions: (**A**) EPCs in monoculture; (**B**) EPCs cocultured with RAW 264.7 macrophages in the absence of nanospheres and without stimuli; (**C**) EPCs cocultured with M2d angiogenic macrophages obtained from RAW 264.7 macrophages treated with 1 μM NECA and 100 ng/mL of *E. coli* LPS; (**D**) EPCs cocultured with RAW 264.7 macrophages in the presence of 50 μg/mL of NanoMBGs; (**E**) EPCs cocultured with RAW 264.7 macrophages in the presence of 50 μg/mL of NanoMBG-IPs. Statistical significance: ### *p* < 0.005, ## *p* < 0.01 (comparisons with EPC RAW).

As can be observed in Figure 6, the coculture of EPCs with control RAW macrophages or M2d angiogenic macrophages produced a decrease or increase in VEGFR2 expression, respectively, when compared to monocultured EPCs. Although these changes were not statistically significant, they are in line with previous results by other authors who demonstrated that anti-inflammatory M2, but not proinflammatory M1 macrophages, promote angiogenesis in vivo [50,51].

The presence of either NanoMBGs or NanoMBG-IPs in the EPC/RAW coculture induced pronounced and significant increases in the percentage of VEGFR2^+^ EPCs, in agreement with results obtained with monocultured EPCs (Figure 5). Again, these data demonstrate the angiogenic effect of this nanomaterial, regardless of the presence of ipriflavone. A nonsignificant increase was observed in the presence of NanoMBG-IPs compared to NanoMBG; however, it appears that, under these coculture conditions, the action of the drug is masked due to the presence of macrophages in the coculture, which also incorporate the nanospheres and do not allow the effect of the drug to be appreciated. Under these experimental conditions, only the angiogenic effect of the nanomaterial itself is evident. These results are in agreement with the data in Figure 5, which shows a similar effect of the conditioned media from macrophages previously treated with both NanoMBGs and NanoMBG-IPs on VEGFR2 expression in EPCs, thus indicating that the presence of the drug in the nanospheres does not change the effect of macrophages on VEGFR2 expression by EPCs.

**Figure 7:**
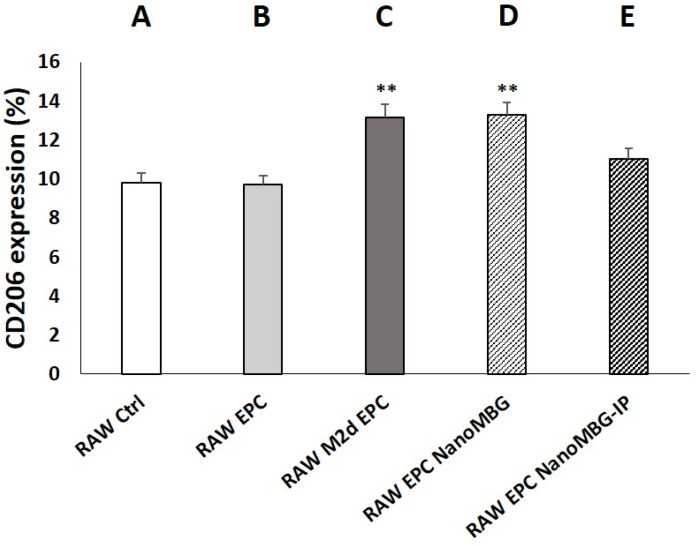
Effects of unloaded (NanoMBGs) and ipriflavone-loaded nanospheres (NanoMBG-IPs) on CD206 expression in RAW 264.7 macrophages cocultured with EPCs. Percentages of CD206^+^ macrophages after 7 days under the following conditions: (**A**) RAW 264.7 macrophages in monoculture; (**B**) RAW 264.7 macrophages cocultured with EPCs in the absence of nanospheres and without stimuli; (**C**) M2d angiogenic macrophages obtained from RAW 264.7 macrophages treated with 1 μM NECA and 100 ng/mL of *E. coli* LPS and cocultured with EPCs; (**D**) RAW 264.7 macrophages cocultured with EPCs in the presence of 50 μg/mL of NanoMBGs; (**E**) RAW 264.7 macrophages cocultured with EPCs in the presence of 50 μg/mL of NanoMBG-IPs. Statistical significance: ** *p* < 0.01 (comparisons with control RAW cells in monoculture).

Figure 7 shows the effect of NanoMBGs and NanoMBG-IPs on CD206 expression in RAW 264.7 macrophages cocultured with EPCs. Although coculture of EPCs with control RAW macrophages did not induce changes in the percentage of CD206^+^ macrophages, when EPCs were cocultured with M2d angiogenic macrophages, a significant increase in this parameter was observed when compared to monocultured control RAW 264.7 cells. This result is consistent with the role of CD206 as a specific marker for M2 macrophage phenotype [40]. On the other hand, the presence of NanoMBGs without IP in the RAW/EPC coculture induced a significant increase of the percentage of CD206^+^ macrophages when compared to control RAW 264.7 cells in monoculture and in coculture with EPCs according to previous studies [23]. The treatment of RAW/EPC cocultures with NanoMBG-IPs also produced an increase in the CD206^+^ percentage, but this was not statistically significant, thus revealing that it is the nanomaterial itself, and not the drug, which induces this marker increase in macrophages. Although the beneficial role of ipriflavone preventing osteoporosis has been evidenced [23,24,25], data on the effects of this drug on macrophages are very scarce. Thus, even when flavonoids can inhibit enzymes or transcription factors relevant in inflammation processes [52], Masilamani et al. indicated that ipriflavone had no effect in the suppression of effector cells of allergies [53]. Further studies are needed to understand how ipriflavone acts on macrophages and other cell types.

## 4. Conclusions

Our study evidences the great potential of unloaded and ipriflavone-loaded mesoporous nanospheres to promote the expression of VEGFR2, directly related to angiogenesis, after their incorporation by endothelial progenitor cells (EPCs). The pathways involved in the entry of these nanospheres into EPCs include clathrin-dependent endocytosis, as the main entry mechanism, as well as phagocytosis and caveolae-mediated uptake. The treatment of EPCs with culture media from basal, M1, and M2 macrophages and studies with cocultures of EPCs with macrophages in the absence and presence of these nanomaterials confirmed the maintenance of their angiogenic effect on EPCs even in the presence of phagocytic cells. Further studies with in vivo models will be conducted to determine the benefits of these nanospheres for bone tissue regeneration via induction of both osteogenesis and angiogenesis.

## Data Availability

Data is contained within the article.
